# Retrospective dosimetric evaluation of the collapsed cone, AAA, and Acuros XB algorithms for lung cancer Halcyon VMAT plans

**DOI:** 10.7717/peerj.20759

**Published:** 2026-02-03

**Authors:** Kainan Shao, Fenglei Du, Lingyun Qiu, Yinghao Zhang, Yucheng Li, Jieni Ding, Wenming Zhan, Weijun Chen

**Affiliations:** 1 Cancer Center, Department of Radiation Oncology, Zhejiang Provincial People’s Hospital (Affiliated People’s Hospital), Hangzhou Medical College, HangZhou, Zhejiang, China; 2 Department of Radiation Physics, Zhejiang Cancer Hospital, Hangzhou Institute of Medicine (HIM), Chinese Academy of Sciences, Hangzhou, Zhejiang, China

**Keywords:** Radiotherapy treatment planning, Halcyon accelerator, Dosimetry, Volumetric modulated arc therapy, Lung cancer radiotherapy

## Abstract

When RayStation is used for Halcyon treatment planning and the plan is transferred to the ARIA/Eclipse system for delivery verification, the dose must be recalculated using the Anisotropic Analytical Algorithm (AAA) or AcurosXB algorithm for compatibility. This study evaluated the dosimetric differences among the Collapsed Cone (CC), AAA, and AcurosXB algorithms for non-small cell lung cancer (NSCLC) volumetric modulated arc therapy (VMAT) plans on the Halcyon platform. Treatment plans for 60 lung cancer patients were initially generated using the CC algorithm in RayStation and then recalculated in Eclipse using AAA and AcurosXB without re-optimization or renormalization. Systematic variations were observed among the three algorithms. AcurosXB showed the largest reductions in target doses compared with CC (up to a 1.56% reduction in clinical target volume (CTV) D2%), while AAA demonstrated smaller differences. For planning target volume (PTV) metrics, both AAA and AcurosXB yielded lower doses than CC (AAA up to 2.16% in D95%; AcurosXB up to 1.58% in D2%). All variations in CTV and PTV metrics remained within approximately 1.7%. For organ-at-risk doses, AAA produced slightly lower values than CC, whereas AcurosXB yielded consistently lower doses across most parameters. Overall, this study shows that AAA and AcurosXB provide slightly lower dose estimates than CC for the same Halcyon plan, especially for PTV and organ-at-risk metrics. These results highlight the importance of consistent dose-calculation methodology in NSCLC radiotherapy, particularly in cross-platform workflows between RayStation and Eclipse.

## Introduction

Radiotherapy plays a critical role in the treatment of lung cancer (Ettinger et al., 2023). For non-small cell lung cancer (NSCLC), a common regimen typically consists of a total dose of 60 Gy, delivered in 30 fractions at 2 Gy per fraction (Cerfolio et al., 2009). Due to the diversity in tumor volume and location within the lung, contemporary solutions employ techniques such as conformal intensity-modulated (IMRT) or volumetric modulated arc therapy (VMAT) (Palma et al., 2008; Holt et al., 2011). Among the available radiotherapy modalities, VMAT has emerged as a key option for NSCLC because of its superior dose distribution, reduced treatment time, and lower radiation exposure to normal tissues (Merrow, Wang & Podgorsak, 2013; Li et al., 2018b). Previous studies have demonstrated that minimizing lung dose parameters such as V_20Gy_ (Graham et al., 1999; Tsujino et al., 2003), V_30Gy_ (Hernando et al., 2001; Piotrowski, Matecka-Nowak & Milecki, 2005), V_5Gy_ (Wang et al., 2006), and the mean lung dose (MLD) (Kwa et al., 1998; Yorke et al., 2002) significantly reduces the risk of radiation pneumonitis (RP). Achieving an optimal balance between adequate target coverage and sparing of normal lung tissue remains a persistent challenge due to variations in tumor size, location, and patient anatomy.

For many years, IMRT and VMAT have been implemented using C-arm accelerators. In 2017, Varian Medical Systems released a new innovation, the ring-mounted Halcyon linear accelerator (Lloyd et al., 2018; De Roover et al., 2019), followed by the 2.0 version in 2019 (Laugeman et al., 2020), which incorporates kilovoltage cone-beam computed tomography (kV-CBCT) and an iterative CBCT reconstruction algorithm (iCBCT) (Jarema & Aland, 2019; Cai et al., 2019). This compact system is equipped with a 6 MV FFF beam capable of rotating the gantry at four rotations per minute (Gao et al., 2019). The Halcyon accelerator introduces several design optimizations over traditional C-arm systems, offering faster image-guided setup and delivery efficiency. Previous studies have confirmed that Halcyon provides comparable or superior plan quality to C-arm accelerators for various treatment sites, including head and neck and cervical cancers, with improved organ-at-risk sparing and workflow efficiency (Li et al., 2018a; Li et al., 2019). In addition, recent investigations on lung cancer VMAT have reported that Halcyon achieves similar or improved low-dose lung sparing (*e.g.*, V_5Gy_) and shorter treatment times compared with conventional systems (Pokhrel et al., 2021; Huang & Liu, 2023). However, only limited evidence has addressed algorithm-related dosimetric discrepancies for lung cancer on the Halcyon platform, particularly regarding the consistency of dose calculation between RayStation and Eclipse planning systems.

Following the introduction of the Halcyon linear accelerator, the majority of radiation therapy planning has been conducted using the Varian Eclipse planning system (Varian Medical Systems, Palo Alto, CA, USA). RayStation (RaySearch Laboratories, Stockholm, Sweden) is a widely utilized third-party radiotherapy planning platform treatment planning system (TPS) (Bodensteiner, 2018). It supports linear accelerators from leading manufacturers such as Varian and Elekta, and it is compatible with TomoTherapy, CyberKnife(Accuray Inc., Sunnyvale, CA, USA) and the Flexitron (Elekta AB, Sweden) brachytherapy system. RayStation (RaySearch Laboratories, Stockholm, Sweden) is a widely utilized third-party radiotherapy planning system (TPS) (Bodensteiner, 2018). It supports linear accelerators from leading manufacturers such as Varian and Elekta, and it is compatible with TomoTherapy, CyberKnife(Accuray Inc., Sunnyvale, CA, USA) and the Flexitron (Elekta AB, Sweden) brachytherapy system. RayStation provides direct machine parameter optimization (DMPO), Python script automation (Yang et al., 2020), and GPU-accelerated collapse cone (CC) dose calculation  (Mzenda et al., 2014; Xu et al., 2014). In our radiotherapy center, RayStation has become the primary platform for treatment planning due to a historical transition from Pinnacle and its use across multiple accelerators, including Trilogy (Varian), TrueBeam, and Infinity (Elekta AB, Sweden). For the sake of workflow consistency, RayStation is often used to optimize and evaluate Halcyon plans. Although Eclipse offers a more integrated solution for Halcyon, the number of purchased Eclipse licenses in our institution is limited, and RayStation therefore remains the routine platform for plan optimization before recalculation in Eclipse for delivery approval. The RayStation 9A version, which supports the dual-layer MLC in Halcyon, has been subject to model creation and assessment studies  (Saini et al., 2021).

However, when a Halcyon treatment plan is approved and transferred to the Varian Aria Record and Verify system for treatment delivery, dose distributions generated by third-party planning systems fail the compatibility (integrity) check. This did not occur on TrueBeam and earlier Linacs because the Aria R&V system requires only the RT plan for delivery. The solution is to recalculate the dose distribution in the Eclipse system while retaining the MU and field weights, using the built-in Anisotropic Analytical Algorithm (AAA) or AcurosXB algorithms.

The collapse cone algorithm used by RayStation and the AAA are both categorized as Type B algorithms, due to their management of lateral electron transport, rather than heterogeneity management. Type A algorithms, such as the pencil beam, account for heterogeneity but do not manage lateral electron transport (Sievinen, Ulmer & Kaissl, 2005; De Martino et al., 2021). In contrast, AcurosXB, a more advanced Type C algorithm, explicitly solves the linear Boltzmann equation to yield results with Monte Carlo-like precision (Akpochafor et al., 2014; Ojala, 2014). Lung tissue, being less dense than other tissues, contributes to the challenges in accurately delivering the prescribed dose to lung tumors, as the lower density can lead to variations in dose distribution, particularly in the regions surrounding tumors. Limited literature has examined clinical planning dose evaluation of RayStation planning with a Halcyon accelerator. In our study on conventional lung cancer therapy, accurate dose calculations considering heterogeneities are vital and indispensable. The dose distributions produced by these three algorithms exhibit slight variations. This research aims to provide a perspective on clinical dose evaluation, serving as a reference for institutions that are using or considering using the RayStation system for Halcyon radiotherapy planning.

## Materials and methods

The study was approved by the Medical Ethics Committee of Zhejiang Provincial People’s Hospital (2024-03-11, No. QT2024053). This study was conducted in accordance with the Declaration of Helsinki, and all research was performed in accordance with relevant guidelines and regulations. Patient consent was waived due to the retrospective and anonymized nature of the analysis. Patients were included if they had pathologically confirmed NSCLC and received conventional radiotherapy between January 1, 2022, and June 1, 2023, with a prescription dose of 60 Gy in 30 fractions, and complete computed tomography (CT) simulation and treatment planning data available. Exclusion criteria included patients who did not receive the prescribed 60 Gy/30 fraction regimen or had incomplete treatment plan or imaging records. All patient data were fully anonymized, and no identifiable information was accessible to the investigators at any stage. Portions of this text were previously published as part of a preprint (https://doi.org/10.21203/rs.3.rs-3493410/v1).

The CT images from 60 NSCLC patients were acquired during their routine clinical radiotherapy workflow. All patients were immobilized in the supine position using thermoplastic masks on an integrated fixation board. CT simulation was performed using a Philips Big Bore Brilliance CT scanner (Philips Medical, Eindhoven, Netherlands). A slice thickness of three mm was used for chest CT acquisition according to institutional protocol, balancing calculation accuracy and clinical workflow efficiency. Most patients underwent free-breathing CT scans with thermoplastic mask immobilization to minimize breathing amplitude, while eight patients underwent 4D CT scanning based on physician evaluation, and their internal target volume (ITV) was generated to account for respiratory motion. The gross tumor volume (GTV), clinical target volume (CTV), and organs at risk (OARs) including the lungs, heart, and spinal cord were delineated following NCCN guidelines and RTOG 0617 recommendations (Bradley et al., 2015). The planning target volume (PTV) was created by uniformly expanding the CTV by 0.5 cm, subject to physician review.

**Figure 1:**
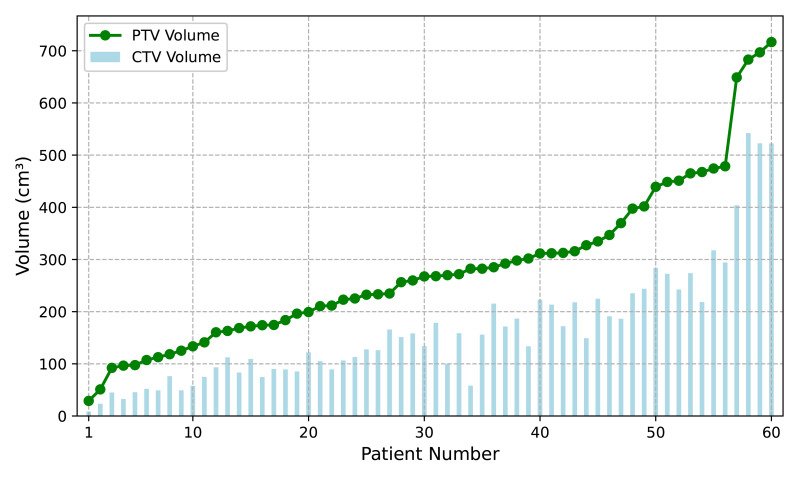
The distribution of CTV and PTV volumes accross 60 patients. The vertical axis represents target volume (cmł), and the horizontal axis shows patient number, sorted by volume of PTV.

This study included 60 patients with pathologically confirmed lung cancer, consisting of 32 patients in stage III (locally advanced disease, 53.3%) and 27 patients in stage IV (metastatic disease, 45%). The anatomical distribution of tumors was as follows: 15 cases (25%) in the left upper lung (LU), 27 cases (45%) in the right upper lung (RU), followed by five cases (8.3%) in the right middle lung (RM), four cases (6.7%) in the left lower lung (LL), and nine cases (15%) in the right lower lung (RL). The mean clinical target volume (CTV) was 165.7 ± 116.89 cmł, and the mean planning target volume (PTV) was 283.38 ± 155.74 cmł. Figure 1 illustrates the volumes of CTV and PTV (*X*-axis: patient number; *Y*-axis: volume in cmł).

All 60 patients received a prescribed radiation dose of 60 Gy, with the prescription dose covering at least 95% volume of the PTV, delivered in 30 fractions over 6 weeks. Treatment plans were designed using RayStation (version 9.0A) and implemented with the Halcyon accelerator using the VMAT technique (also called RapidArc by Varian). To protect the normal lung tissue and minimize the low-dose region, all patients were treated using the partial arcs mentioned (182–230, 30–60, 130–178) along with the counter-clockwise complementary arcs (total of 6 arcs). The collimator angle was selected based on the patient’s anatomy, with a choice of 10° or 350° depending on the direction of the long axis of the PTV in the beam-eye-view.

The clinical evaluation criteria for the treatment plan’s target volumes and OAR were derived from the NCCN guidelines and the RTOG 0617 protocol and further tailored to the clinical practices of our institution. The plan optimization employed the Direct Machine Parameter Optimization (DMPO) algorithm to utilize a progressive semiautomatic optimization strategy (Yang et al., 2020). The clinical goals for the Halcyon plans includes the following key parameters: PTV coverage of 6,000 cGy (D95% ≥ 60 Gy), maximum dose (D0.1cc) < 6,900 cGy (115%), and a conformity index (CI) > 0.8. For the lungs, constraints include V20Gy < 28%, V30Gy < 18%, and V5Gy < 46%, with a mean dose of < 1,200 cGy. The heart is limited to a mean dose of < 2,500 cGy, while the spinal cord’s maximum dose (D0.03cc) is constrained to < 4,500 cGy. The maximum dose point must fall within the range of PTV, and normal tissue constraints aim to minimize dose as much as possible. For clinical plan evaluation, the variable “VxGy” is defined as the volume of a structure receiving greater than or equal to x Gy, and “Dxcc” is defined as the largest dose received by *x*cm^3^ of the structure. The maximum dose of a structure is defined by D0.03cc, which represents the dose delivered to the smallest 0.03 cubic centimeter of the structure. Equivalent uniform dose (EUD) (Niemierko, 1997) functions were used in the optimization process (Wu et al., 2002). The Paddick Conformity Index (CI) (Paddick, 2000) was calculated using the formula *CI* = (*TV*_*PIV*_)^2^/(*TV*∗*PIV*), where *TV*_*PIV*_ is the target volume covered by the 60 Gy prescription isodose volume, *TV* is the volume of the target, and *PIV* is the volume of the prescription isodose. The homogeneity index (HI) was calculated as *HI* = (*D*_2%_ − *D*_98%_)/*D*_50%_, following the definition recommended in ICRU Report 83 (Hodapp, 2012), to quantify the uniformity of the PTV dose distribution.

Clinical dose calculations were performed using the collapsed cone (CC) algorithm version 5.1 on the RayStation 9A, with a dose grid resolution of 0.25 cm in all three directions. The computation times ranged from ten to twenty seconds. For consistency, the treatment table structures (Couch Interior and Couch Surface) were imported from Aria Eclipse 15.6 for each patient, ensuring that the positions and CT values (density values) of these ROIs remain consistent between the Eclipse and RayStation systems. Both plan optimization and evaluation accounted for the influence of the treatment couch. The CT density table used in this study was generated using an electron density phantom model (062M) on our treatment planning CT simulator. This table includes the conversion between CT numbers (HU), mass density (MD), and relative electron density (RED) for each tissue material. The Collapsed Cone (CC) and AcurosXB (AXB) algorithms use CT numbers to derive mass density, while the Analytical Anisotropic Algorithm (AAA) uses the CT values to derive RED. The same CT–MD–RED table is used in both RayStation and Eclipse to ensure consistency in the conversion process. After the treatment plan was finalized, it was reviewed and approved by the radiation oncologist and medical physicist. The plan was transferred to the Aria Record and Verify (R&V) system. In the Aria Eclipse system, the volume dose was calculated using both the AAA (version 15.6) and AcurosXB (version 15.6) algorithm with a 0.25 cm grid, while the MU and other beam parameters were unchanged. For a CT dataset with a three mm slice thickness in Eclipse, the resolution was 0.25 cm in the X and Y directions, and 0.3 cm in the Z-direction. This was facilitated using the ”calculate volume with preset value” feature. According to the recommendations by Kry et al. (2021), The calculation reporting mode for AcurosXB is dose-to-medium, because this mode best represents the true dose based on tissue composition. The results were then reimported into RayStation for dose evaluation and comparison.

The clinical evaluation criteria were collected based on doses calculated for 60 patients using the CC, AAA, and AcurosXB algorithms, referred to as the haCC, haAAA, and haAXB groups, respectively. Continuous variables are presented as the mean ± standard deviation (Mean ± SD). The Shapiro–Wilk test was used to assess the normality of the paired differences between algorithms. Because the dose metrics from different algorithms were compared within the same patient cohort, forming paired datasets, the paired *t*-test was applied for normally distributed paired differences, while the Wilcoxon signed-rank test was used as the appropriate non-parametric alternative when normality was not satisfied. Pairwise comparisons were performed between the haCC and haAAA groups, as well as between the haCC and haAXB groups. This approach minimizes inter-patient variability by ensuring that each pairwise comparison is based on identical beam geometry and patient anatomy. To control the family-wise error rate across these two planned comparisons, the Bonferroni correction (Armstrong, 2014 was applied) therefore, the significance threshold was adjusted to *p* = 0.025 (0.05/2). All statistical analyses were performed using SciPy (Python 3.10).

## Results

**Figure 2:**
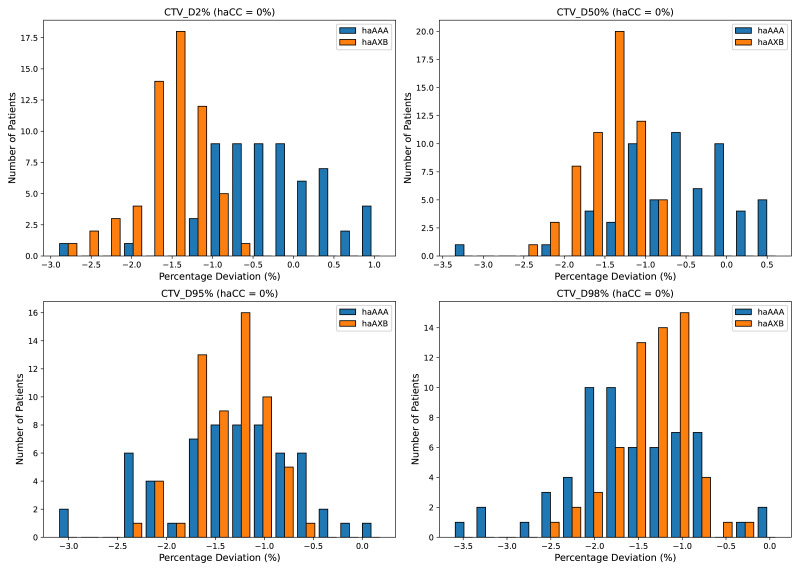
Histograms illustrating the deviation in dose metrics for the clinical target volume (CTV) between the CC, AAA, and AcurosXB algorithms. The deviations of groups haAAA and haAXB are shown as percentages for D2%, D50%, D95%, and D98%, calculated using the formula (*haAAA* − *haCC*)/*haCC* × 100% and (*haAXB* − *haCC*)/*haCC* × 100%, with haCC as the reference (0% deviation).

In the CTV assessment, the dosimetric analysis revealed systematic variations between the planning algorithms. Specifically, the AAA algorithm yielded marginally lower values for the dose metrics, with the D2%, D50%, D95%, D98%, and mean dose showing average decreases of 0.29%, 0.61%, 1.36%, 1.62%, and 0.68%, respectively, compared to the CC algorithm. A more pronounced difference was observed when employing the AcurosXB algorithm, which resulted in average reductions of 1.56%, 1.46%, 1.39%, 1.36%, and 1.47% across the same dose parameters. These differences indicate that the AcurosXB algorithm produces greater deviations compared to the CC algorithm, especially in CTV D98% and CTV D95%, though the differences in these CTV metrics do not exceed approximately 1.5%. Figure 2 illustrates the CTV dose distributions using histograms, showing the deviations between the different algorithms (The deviation is calculated as (*haAAA* − *haCC*)/*haCC* × 100%.)

**Figure 3:**
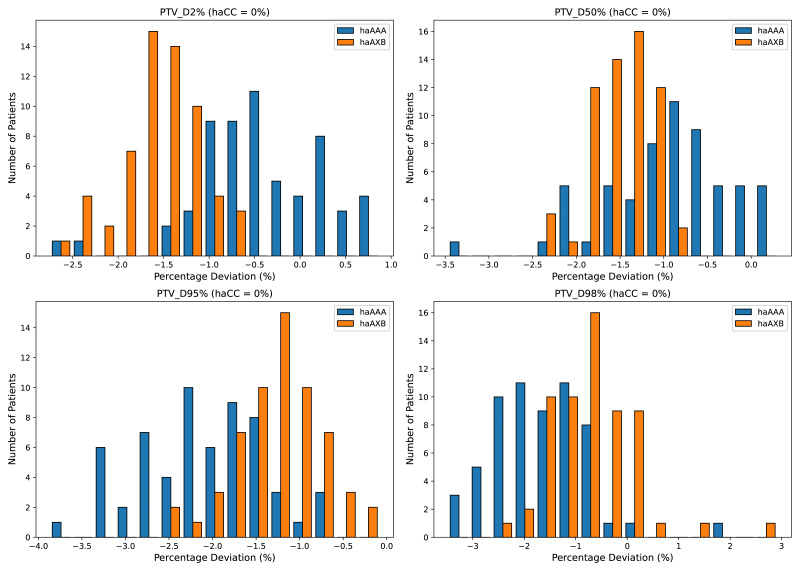
Histograms illustrating the deviation in dose metrics for the planning target volume (PTV) between the CC, AAA, and AcurosXB algorithms. The deviations of groups haAAA and haAXB are shown as percentages for D2%, D50%, D95%, and D98%, calculated using the formula (*haAAA* − *haCC*)/*haCC* × 100% and (*haAXB* − *haCC*)/*haCC* × 100%, with haCC as the reference (0% deviation).

Similar trends were observed for the PTV, with the AAA algorithm showing decreases of 0.41%, 0.95%, 2.16%, 1.68%, and 1.03% across the D2%, D50%, D95%, D98%, and mean dose metrics, respectively, whereas the AcurosXB algorithm showed average decreases of 1.58%, 1.50%, 1.25%, 0.70%, and 1.44%, respectively (Fig. 3).

**Figure 4:**
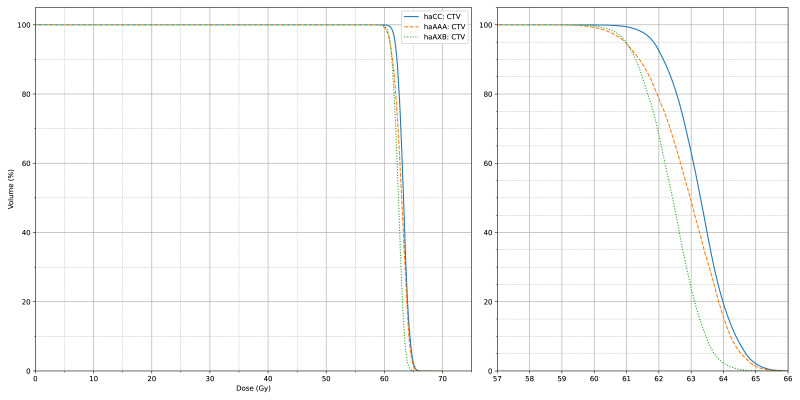
The average CTV dose-volume histogram (DVH) curves of haCC, haAXB and haAAA for 60 patients. The right panel shows an enlarged view of the main figure, focusing on the *x*-axis range from 57 to 66 Gy.

**Figure 5:**
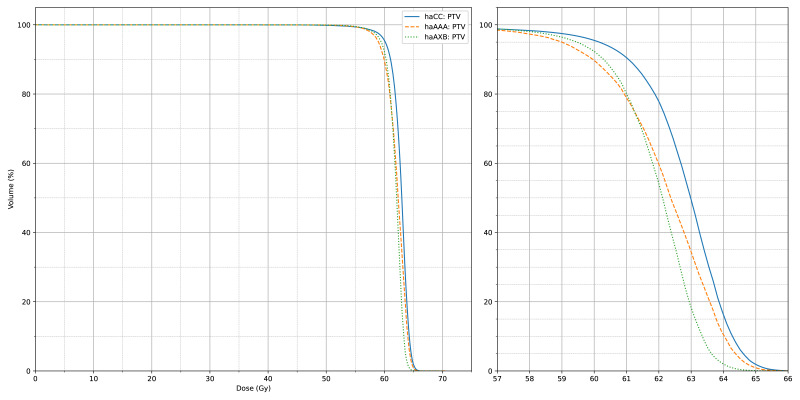
The average PTV dose-volume histogram (DVH) curves of haCC, haAXB and haAAA for 60 patients. The right panel shows an enlarged view of the main figure, focusing on the *x*-axis range from 57 to 66 Gy.

Figures 4 and 5 also display the average dose-volume histogram (DVH) curves for the 60 patients, comparing haCC, haAXB, and haAAA groups. The DVH curves highlight distinct patterns: in high-dose regions of the target (*e.g.*, D2%), haCC results closely aligned with haAAA, whereas in lower dose regions (*e.g.*, D95%), the dose distribution of haCC was more similar to that of haAXB. When using PTV D95% as a reference for equivalent prescription dose, haAXB demonstrated greater agreement with haMC in terms of dose coverage than haAAA.

**Figure 6:**
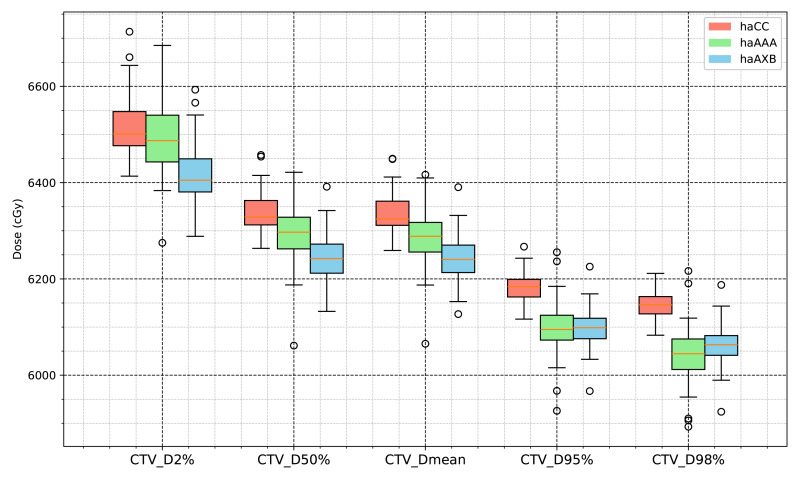
Boxplot comparison of dose metrics for clinical target volume (CTV) across the three algorithms: haCC (red), haAAA (green), and haAXB (blue).

**Figure 7:**
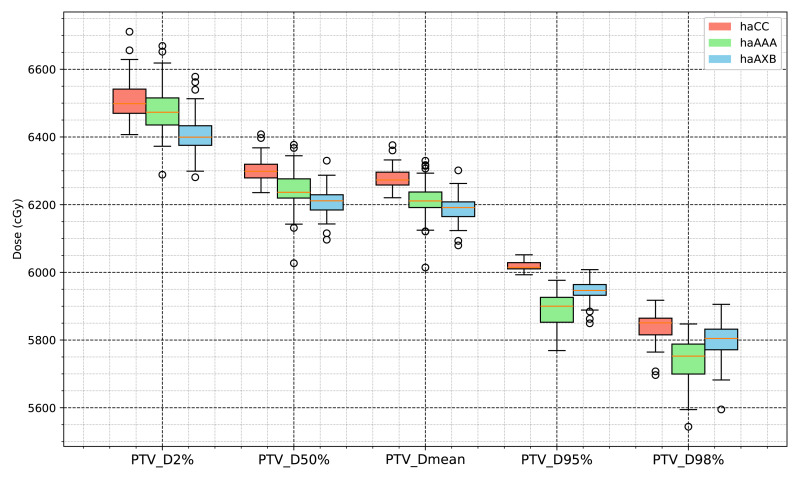
Boxplot comparison of dose metrics for planning target volume (PTV) across the three algorithms: haCC (red), haAAA (green), and haAXB (blue).

The statistical analysis results for the target-related dose indices are presented in Table 1 and Figs. 6 and 7. The CI for the PTV varied with the planning algorithms, with the haCC algorithm resulting in a CI of 0.841, which is slightly higher than the 0.795 obtained with the haAAA algorithm, while the haCC and haAXB algorithms showed similar CI values of 0.841 and 0.847, respectively. Differences in target dose metrics can also lead to corresponding differences in the calculated homogeneity index (HI). The HI values for haCC, haAAA, and haAXB were 0.107, 0.119, and 0.099, respectively.

**Table 1:** Comprehensive dose evaluation parameters for target volumes using different planning algorithms. CI is calculated following the Paddick Conformity formula with respect to PTV and 60Gy. HI is calculated as *HI* = (*D*_2%_ − *D*_98%_)/*D*_50%_ with respect to PTV dose metrics.

Evaluations	haCC	haAAA	*P* value (haCC *vs* haAAA)	haAXB	*P* value (haCC *vs* haAXB)^1^
CTV D2% (cGy)	6,514.1 ± 60.1	6,495.0 ± 78.5	0.0028	6,414.2 ± 60.5	<0.001
CTV D50% (cGy)	6,335.5 ± 40.7	6,297.2 ± 64.2	<0.001	6,244.1 ± 45.8	<0.001
CTV D95% (cGy)	6,181.7 ± 28.2	6,098.5 ± 54.8	<0.001	6,096.8 ± 37.0	<0.001
CTV D98% (cGy)	6,143.2 ± 28.7	6,045.5 ± 57.0	<0.001	6,060.6 ± 39.1	<0.001
CTV Dmean (cGy)	6,333.5 ± 39.8	6,290.6 ± 61.8	<0.001	6,242.0 ± 44.8	<0.001
PTV D2% (cGy)	6,509.5 ± 58.4	6,483.0 ± 73.9	<0.001	6,408.5 ± 58.3	<0.001
PTV D50% (cGy)	6,301.1 ± 34.5	6,242.1 ± 59.5	<0.001	6,208.0 ± 40.1	<0.001
PTV D95% (cGy)	6,019.1 ± 14.1	5,892.0 ± 46.4	<0.001	5,944.4 ± 30.6	<0.001
PTV D98% (cGy)	5,838.0 ± 43.0	5,741.5 ± 61.6	<0.001	5,797.7 ± 53.5	<0.001
PTV Dmean (cGy)	6,276.3 ± 31.6	6,212.3 ± 53.6	<0.001	6,187.2 ± 38.3	<0.001
(PTV) HI	0.107 ± 0.012	0.119 ± 0.014	<0.001	0.099 ± 0.011	<0.001
(PTV) CI	0.841 ± 0.043	0.795 ± 0.055	<0.001	0.847 ± 0.038	0.0106

**Notes.**

1 The *P* values signify statistical significance, with a value of <0.001 indicating a highly significant difference between the algorithms.

In the evaluation of organ-at-risk doses, significant differences were observed among the haCC, haAAA, and haAXB algorithms across various dose metrics. For volume dose metrics such as Lungs V5Gy, Lungs V10Gy, Lungs V20Gy, and Lungs V30Gy, statistically significant differences were found between the haCC and haAAA algorithms, with haAAA generally delivering slightly higher doses compared to haCC. For example, in Lungs V5Gy, the dose for haAAA was 36.5% (±8.4), higher than haCC at 35.7% (±8.1), with a difference of 0.72% (*p* < 0.001). In comparison to haCC, the haAXB algorithm consistently delivered lower doses, especially in dose mean metrics such as Lungs Dmean and Lungs-PTV Dmean, where haAXB showed a noticeable reduction in dose. For instance, Lungs Dmean was 1017.1 cGy (±258.6) for haCC, while haAXB was 1003.9 cGy (±257.1), a difference of 13.2 cGy (*p* < 0.001). Similarly, dose differences in organs such as the Heart and SpinalCord also demonstrated significant variability, with haAXB particularly showing lower doses to the heart and spinal cord. The comparison of Body D0.03cc doses between the algorithms reveals that haAAA results in a slight decrease compared to haCC, with a difference of 19.4 cGy (*p* = 0.0139), while haAXB shows a more pronounced reduction of 88.5 cGy (*p* < 0.001) compared to haCC.

**Figure 8:**
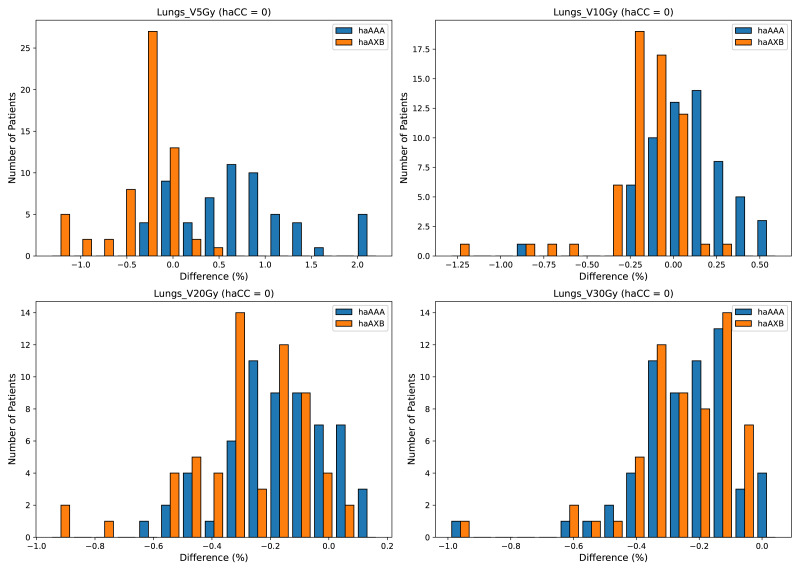
Histograms illustrating the deviation in dose metrics dose-volume indices for the lungs, including V5Gy, V10Gy, V20Gy, and V30Gy, between the CC, AAA, and AcurosXB algorithms. The differences between groups are calculated as *haAAA* − *haCC* and *haAXB* − *haCC*.

**Figure 9:**
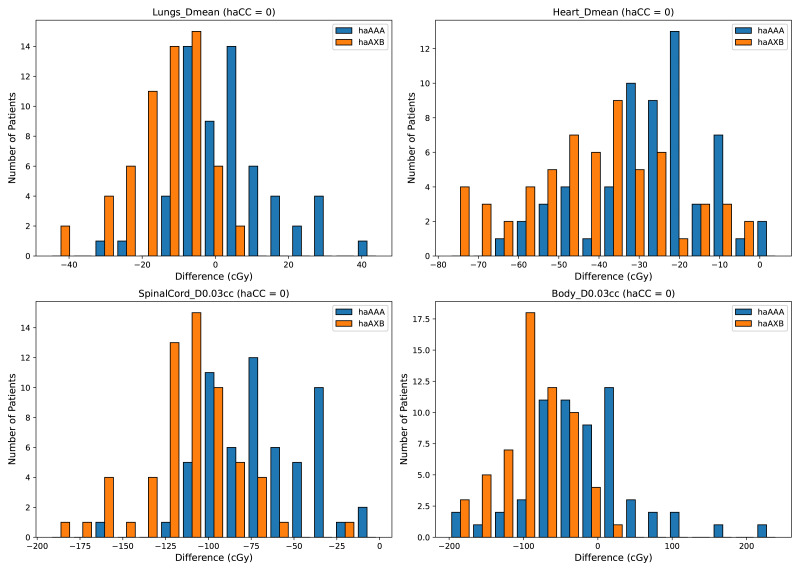
Histograms illustrating the deviations in dose metrics, including dose-volume indices for the mean doses of the lungs and heart, and D0.03cc for the SpinalCord and Body, between the CC, AAA, and AcurosXB algorithms. The differences between groups are calculated as *haAAA* − *haCC* and *haAXB* − *haCC*.

**Figure 10:**
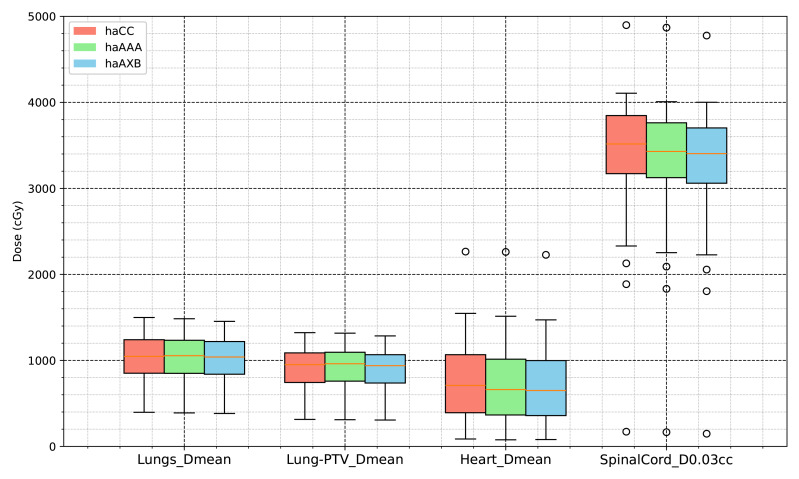
Comparative analysis of dose distribution statistics for organs at risk using the AAA and CC and AcurosXB algorithms.

**Figure 11:**
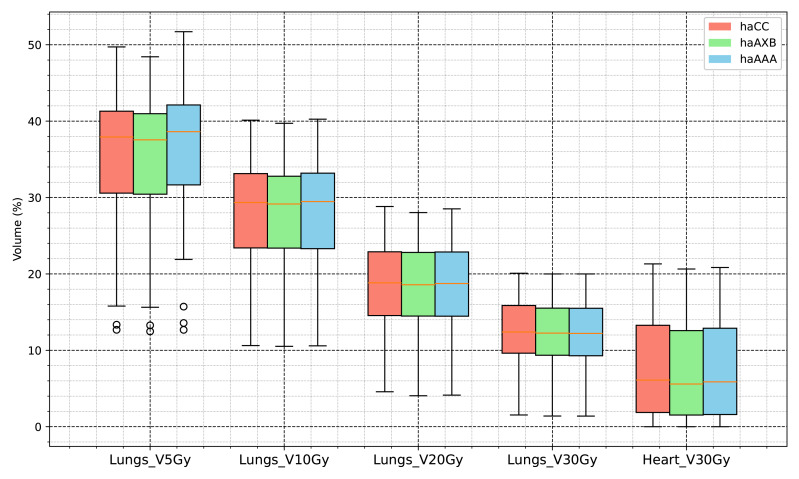
Comparative analysis of dose histogram statistics for organs at risk using the AAA and CC and AcurosXB algorithms.

A detailed comparison of dose indices for organs at risk is provided using histograms, showing the differences between the different algorithms, with haCC used as the reference (*i.e.,* the differences are calculated as haAAA - haCC and haAXB - haCC). Figure 8 presents the dose-volume indices for the lungs, including V5Gy, V10Gy, V20Gy, and V30Gy, while Fig. 9 illustrates the dose values for Lungs Dmean, Heart Dmean, SpinalCord D0.03cc, and Body D0.03cc. The statistical analysis results for the dose indices of organs at risk are presented in Table 2 and Figs. 10 and 11. In addition, the total MU was 706.7 ± 120.2, corresponding to a modulation factor of 3.53 ± 0.60 MU/cGy. The delivery time for each patient was 56.6 ± 10.9 s, which is approximately equivalent to the MU divided by 800 MU/min dose rate, indicating that the Halcyon system always operated at the maximum dose rate.

## Discussion

In this study, we quantitatively compared the CC, AAA, and AcurosXB algorithms for Halcyon-based VMAT plans in 60 patients with NSCLC. Our findings showed systematic but relatively small inter-algorithm differences. For target coverage, AAA and AcurosXB produced slightly lower doses than CC, with reductions of approximately 1–2% across most CTV and PTV metrics. For OARs, AcurosXB yielded the lowest doses, followed by AAA, while CC resulted in the highest values. These trends are consistent with the dose–calculation characteristics of the three algorithms and with observations reported in previous studies.

The observed pattern can be explained by the underlying physical modeling of each algorithm. Both CC and AAA are Type B convolution–superposition algorithms that manage lateral electron transport but rely on simplified handling of tissue heterogeneity. As a result, they tend to overestimate dose in low-density lung regions, as previously reported by Kry et al. (2013) and Zhen et al. (2015). In contrast, AcurosXB is a Type C algorithm that explicitly solves the linear Boltzmann transport equation, providing dose distributions that more closely approximate Monte Carlo (MC) calculations (Ojala, 2014). This behavior explains why AcurosXB consistently produced lower and more physically realistic doses in our cohort.

Monte Carlo simulations are widely regarded as the reference standard for heterogeneous media such as lung. Kry et al. (2013) demonstrated that CC and AAA overestimated thoracic phantom doses by approximately 3.5%, whereas MC agreed with measurements within 0.6%. For Halcyon specifically, Laakkonen et al. (2023) recently validated a Monte Carlo model, confirming its superior accuracy. Compared with these reports, the inter-algorithm differences observed in our study (mostly within 1–1.6%) are smaller, likely because our cohort involved conventional fractionated NSCLC plans rather than high-gradient Stereotactic Ablative Radiotherapy (SABR) treatments, where heterogeneity effects are more pronounced.

These findings have practical implications for clinical workflow. The Halcyon beam model in RayStation and the CC algorithm used in this study were fully commissioned by senior clinical physicists at our institution and are approved for routine use in treatment planning and optimization. All plans generated with RayStation undergo standard clinical review by both radiation oncologists and physicists before being archived in the patient’s treatment record. When Halcyon plans optimized in RayStation using the CC algorithm are recalculated in Eclipse for treatment delivery, the slight systematic reductions in dose predicted by AAA or AcurosXB may lead to marginally lower PTV coverage. For centers relying on such cross-platform workflows, a small normalization adjustment in RayStation may be considered to maintain consistent PTV D95% after recalculation, although this approach is not mandatory and should be applied cautiously.^1^ 1For example, increasing the RayStation normalization to approximately 101% (*e.g.*, prescribing 60.6 Gy instead of 60 Gy) can help ensure that at least 95% of the PTV receives 60 Gy when recalculated with AcurosXB in Eclipse, which is recognized as being closer to Monte Carlo accuracy.In addition, AcurosXB may be preferred when high accuracy is required in cases involving complex lung heterogeneity.

This study has several limitations. The sample size was moderate, and only conventional fractionation plans were evaluated. The potential interplay between tumor location, motion amplitude, and algorithmic differences could not be fully assessed and warrants further investigation with a larger cohort. Although Monte Carlo was not included in the clinical comparison, future work will incorporate a clinically commissioned MC algorithm for more definitive validation of algorithm performance.^2^ 2Due to manuscript scheduling and publication timelines, the Monte Carlo comparison study has since been completed and published (Shao et al., 2025).

**Table 2:** Dose evaluation parameters for organs at risk with comparative analysis across planning algorithms.

Evaluations	haCC	haAAA	*P* value (haCC *vs* haAAA)	haAXB	*P* value (haCC *vs* haAXB)^1^
Lungs V5Gy (%)	35.7 ± 8.1	36.5 ± 8.4	<0.001	35.4 ± 8.0	<0.001
Lungs V10Gy (%)	27.7 ± 6.8	27.8 ± 6.9	0.0013	27.6 ± 6.8	<0.001
Lungs V20Gy (%)	18.4 ± 5.6	18.3 ± 5.7	<0.001	18.2 ± 5.6	<0.001
Lungs V30Gy (%)	12.3 ± 4.5	12.0 ± 4.5	<0.001	12.0 ± 4.5	<0.001
Lungs Dmean (cGy)	1,017.1 ± 258.6	1,021.2 ± 259.3	0.0234	1,003.9 ± 257.1	<0.001
Lungs-PTV Dmean (cGy)	907.4 ± 239.8	914.8 ± 241.4	<0.001	895.6 ± 238.3	<0.001
Heart V30Gy (%)	7.5 ± 6.3	7.1 ± 6.1	<0.001	7.0 ± 6.0	<0.001
Heart Dmean (cGy)	744.4 ± 455.5	716.9 ± 452.3	<0.001	703.6 ± 443.2	<0.001
SpinalCord D0.03cc (cGy)	3,396.6 ± 673.9	3,326.6 ± 665.6	<0.001	3,285.1 ± 658.5	<0.001
Body D0.03cc (cGy)	6,641.8 ± 77.8	6,622.4 ± 104.8	0.0139	6,553.3 ± 68.5	<0.001

**Notes.**

1 *P* value represents the statistical comparison between haCC and the other algorithms.

## Conclusion

This study demonstrates consistent and systematic differences in dose calculations among the CC, AAA, and AcurosXB algorithms when applied to Halcyon VMAT plans for lung cancer. Compared with RayStation’s CC algorithm, recalculation in Eclipse using AAA or AcurosXB results in slightly lower target and organ-at-risk doses, with AcurosXB showing the greatest reductions due to its more advanced modeling of tissue heterogeneity.

These findings indicate that algorithm selection can meaningfully influence dose evaluation in cross-platform clinical workflows. AcurosXB may be preferred in scenarios requiring higher dosimetric accuracy in low-density lung regions. When RayStation-generated plans are recalculated in Eclipse for treatment delivery, radiation oncologists and physicists should be aware of the expected dose reductions, and may consider minor normalization adjustments if clinically appropriate to maintain consistent PTV coverage.

Overall, this work provides practical guidance for improving consistency in Halcyon-based radiotherapy planning across different treatment planning systems and highlights the importance of understanding algorithm-specific behavior in clinical decision-making.

## Supplemental Information

10.7717/peerj.20759/supp-1 Supplemental Information 1 AXB data

10.7717/peerj.20759/supp-2 Supplemental Information 2 CC data

10.7717/peerj.20759/supp-3 Supplemental Information 3 AAA data

10.7717/peerj.20759/supp-4 Supplemental Information 4 STROBE Checklist

## Abbreviations

AAA: Analytical Anisotropic Algorithm
AXB: AcurosXB
CC: Collapsed Cone
CTV: Clinical Target Volume
PTV: Planning Target Volume
NSCLC: Non-Small Cell Lung Cancer
IMRT: Intensity-Modulated Radiation Therapy
VMAT: Volumetric-Modulated Arc Therapy
CT: Computed Tomography
CBCT: Cone Beam Computed Tomography
OAR: Organs At Risk
MLC: Multi-Leaf Collimator
IGRT: Image-Guided Radiation Therapy
TPS: Treatment Planning System
DMPO: Direct Machine Parameter Optimization
DVH: Dose-Volume Histogram
EUD: Equivalent Uniform Dose
CI: Conformity Index
RTOG: Radiation Therapy Oncology Group

## Additional Information and Declarations

### Data Availability

The following information was supplied regarding data availability: The data are available in the Supplemental Files.
